# Presentations of adult septic patients in the prehospital setting as recorded by emergency medical services: a mixed methods analysis

**DOI:** 10.1186/s13049-017-0367-z

**Published:** 2017-03-03

**Authors:** Ulrika Margareta Wallgren, Katarina Eva Margareta Bohm, Lisa Kurland

**Affiliations:** 1 Karolinska Institutet, Department of Clinical Science and Education, Södersjukhuset, Sjukhusbacken 10, SE 118 83 Stockholm, Sweden; 2 Fisksätra Vårdcentral (Primary Health Care Center), Fisksätra torg 20, SE 133 41 Saltsjöbaden, Sweden; 3 Department of Medical Sciences, Örebro University, SE 701 82 Örebro, Sweden

**Keywords:** Emergency Medical Services, Prehospital, Emergency Care, Sepsis

## Abstract

**Background:**

Current sepsis screening tools rely on vital parameters which are, however, normal in one third of patients with serious infections. Therefore, there is a need to include other variables than vital parameters to identify septic patients. Our primary aim was to identify and quantify keywords related to the septic patients’ symptom presentation in the prehospital setting. The secondary aims were to compare keywords in relation to in-hospital mortality and the distribution of keywords in relation to age categories, survivors/ deceased and severe/ non-severe sepsis.

**Methods:**

A mixed methods analysis using a sequential exploratory design was performed, starting with a content analysis of presentations of septic patients as documented in Emergency Medical Services (EMS) records (*n* = 80) from 2012, to identify keywords related to sepsis presentation. Thereafter, the identified keywords were quantified among 359 septic patients from 2013. All patients were adults, admitted to Södersjukhuset and discharged with an ICD-10-code (International Classification of Diseases, Tenth Revision) compatible with sepsis.

**Results:**

The most common keywords related to septic patients’ symptom presentation were: abnormal/ suspected abnormal temperature (64.1.%), pain (38.4%), acute altered mental status (38.2%), weakness of the legs (35.1%), breathing difficulties (30.4%), loss of energy (26.2%) and gastrointestinal symptoms (24.0%). There was an association between keywords and in-hospital mortality. Symptoms varied between age categories, survivors/ deceased and severe/ non-severe sepsis.

**Discussion:**

This is, to the best of our knowledge, the first study exploring the symptom presentation as documented by EMS, of septic patients in the prehospital setting. Keywords related to patients´ symptom presentation recurred in the EMS records of septic patients, so that a pattern was discernible. In addition, certain symptom presentations were associated with increased in-hospital mortality

**Conclusions:**

Information relating to symptom presentation is not included in current sepsis screening tools. We suggest that keywords related to patients´ symptom presentation could be integrated into screening tools and may thus increase the identification of sepsis, and potentially also identify high-risk patients. However, as a first step, the specificity of these keywords, with respect to sepsis, needs to be examined.

**Electronic supplementary material:**

The online version of this article (doi:10.1186/s13049-017-0367-z) contains supplementary material, which is available to authorized users.

## Background

Sepsis, caused by a dysregulated host response to infection [1], is one of the most important conditions to identify within emergency care due to its high mortality and to a large extent treatable cause.

The mortality of severe sepsis (19–30%) [2, 3] is more than three times higher than that of myocardial infarction (6–8%) [4, 5], and rapid identification and therapy has traditionally been thought to be associated with improved outcome [6–9]. However, the systematic review and meta-analysis by Sterling et al. [10] questioned the benefit of early antibiotic treatment. Nevertheless, a recalculation by Yokee et al. questioned these conclusions [11] and the recommendation of early antibiotic treatment remains a recommendation [12].

Sepsis is a clinical diagnosis which can be defined as the presence of an infection in combination with two or more SIRS (Systemic Inflammatory Response Syndrome) criteria [13, 14]. SIRS is in turn based mainly on vital parameters. However, 39% of the patients with serious infections lack abnormal vital parameters [15] and 12% of the patients with severe sepsis do not fulfil the SIRS criteria [16]. The inadequate sensitivity and specificity of the SIRS criteria has been a contributing factor to a recently suggested revision of the sepsis definition [1]. Nevertheless, existing sepsis screening tools are still based mainly on SIRS criteria [17, 18].

The diagnostic and prognostic significance of medical history is incompletely known regarding sepsis [19]. Our hypothesis is that inclusion of variables related to septic patients’ reported symptom presentation may add value to a future screening tool.

The primary aim of the current study was to explore the presentations of adult septic patients in the prehospital setting as documented in EMS medical records and to identify and quantify keywords related to septic patients’ symptom presentation according to EMS documentation. The secondary aims were to compare keywords in relation to in-hospital mortality and the distribution of keywords in relation to age categories, survivors/ deceased and severe/ non-severe sepsis.

## Methods

### Study design and setting

This is a mixed methods analysis [20, 21] of adult patients arriving by EMS to Södersjukhuset through the ED and discharged with an ICD-10-code compatible with sepsis. The mixed methods analysis [20, 21] combines qualitative methods and quantitative methods and in the current study the sequential exploratory design [20] was used, starting with a content analysis [22, 23] of patients admitted during 2012. The content analysis was performed on the content of the narrative section of the EMS records, where presentations of adult septic patients in the prehospital setting are described, and served to identify keywords related to sepsis presentation. Second, the keywords identified in the content analysis were quantified in a separate cohort of septic patients admitted during 2013. For a description of selected cases, see “Selection of study participants and data collection”.

The Stockholm EMS transports approximately 200,000 patients annually and serves both rural and urban areas. The furthest road distance to hospital within the catchment area is 70–75 km. Ambulances are typically staffed with a nurse specialist and a paramedic. The patients in the study were admitted to Södersjukhuset which is an urban, 704-bed teaching hospital with approximately 128,000 adult Emergency Department (ED) visits in 2015 [24].

### Selection of study participants and data collection

Adult patients (18 years old or above), arriving by the EMS to Södersjukhuset through the ED and discharged from in-hospital care with an ICD-10-code compatible with sepsis [25] (including septic arthritis) were candidates for inclusion.

Patients admitted during 2012 were included in the content analysis and patients admitted during 2013 were included for quantification of the identified keywords. Medical records were obtained through the in-hospital record system (Pasett, Sweden, Version 1.61).

#### Content analysis of patients admitted during 2012

The maximum variation sampling method [26] was used for inclusion of patients to the content analysis of patients admitted during 2012, to achieve maximum diversion regarding arrival time, gender, season and age as these factors could affect the presentations of the patients. The first and the last male and female patient every month within the following age categories: <65 years, 65–74 years and 75 years or older [27] were included. To obtain diversion over day and night, patients that arrived daytime (>8:00 am - ≤20:00 pm) were included uneven months and patients that arrived at night (>20:00 pm - ≤8:00 am) were included even months. The aim was to include patients until the point where collecting additional data did not yield new information [28], a condition referred to as “saturation” within qualitative research [28, 29]. There is no commonly accepted sample size for qualitative studies, as it depends on richness of data [28]. In the current study we obtained no additional information after approximately 50 EMS records had been analyzed in the content analysis of patients admitted during 2012, but continued to analyze a total of 80 records in accordance with previously published analyzes of medical records [30, 31].

#### Quantification of keywords among patients admitted during 2013

**Fig. 1:**
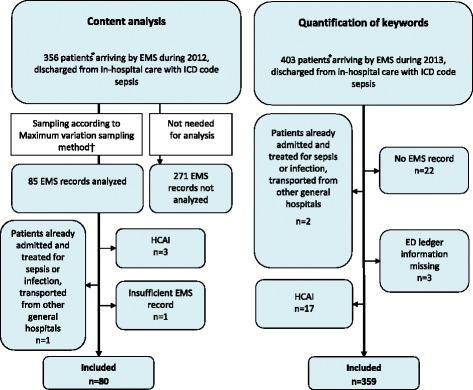
Flow chart for inclusion and exclusion. Flow chart for inclusion and exclusion of patients in the content analysis of septic patients arriving by EMS and admitted to Södersjukhuset through the ED during 2012 and the quantification of the keywords among septic patients arriving by EMS and admitted to Södersjukhuset through the ED during 2013, respectively. EMS = Emergency Medical Services, ED = Emergency Department, HCAI = Health Care Associated Infection, ICD-10 = International Classification of Diseases, 10:th Revision

Inclusion of at least 350 patients admitted during 2013 was required for the quantification of keywords among patients admitted during 2013, in accordance with our sample size calculation; assuming a documented relative frequency of 50% for individual keywords, 350 patients would render a 95% confidence interval of ± 5%. However, all 403 EMS patients admitted through the ED and discharged with ICD code sepsis during 2013 were screened, and 359 included, since the amount of patients fulfilling eligibility criteria during 2013 just barely exceeded this number. See Fig. 1.

#### Exclusion criteria

Exclusion criteria both 2012 and 2013 were: Healthcare-Associated Infections (HCAI) defined as onset of infection ≥48 h after ED admission [32], subjects already admitted and treated for sepsis or infections transported from other general hospitals, EMS records with insufficient information, lack of EMS records and patients with no information in the electronical ED ledger (AkuSys, Sweden, Version 5.5b). See Fig. 1, Flow chart for inclusion and exclusion of patients.

### Definitions

This study was performed prior to the proposed introduction of a new sepsis definition [1], and the terms severe and non-severe sepsis [13, 14, 33] are used throughout the article.

The definition of severe sepsis is described in Additional file 1.

Deceased was defined as in-hospital death in accordance with the in-hospital record system Pasett.

### Outcomes

Our primary outcome was the prevalence of keywords related to septic patients’ symptom presentation according to EMS documentation. Secondary outcomes were in-hospital mortality and the distribution of keywords in relation to age categories, survivors/ deceased and severe/ non-severe sepsis.

### Analysis

#### 1. Content analysis of patients admitted during 2012

An inductive manifest content analysis of patients admitted during 2012 inspired by Krippendorff [23] was performed on the narrative section of the EMS records. This section contains a description of both the patient’s symptom presentation as well as brief descriptions of physical findings. The narrative section mainly reflects information reported to the EMS by the patient/ relatives/ bystanders/ personnel at other health care facilities such as nursing homes, as well as the general impression achieved by the EMS personnel. It is not always possible to trace the source of documented statements, i.e., to differentiate between whether the information comes from the patient, relatives/ bystanders or the EMS personnel. Vital parameters are registered in a separate part of the EMS record.

**Fig. 2:**
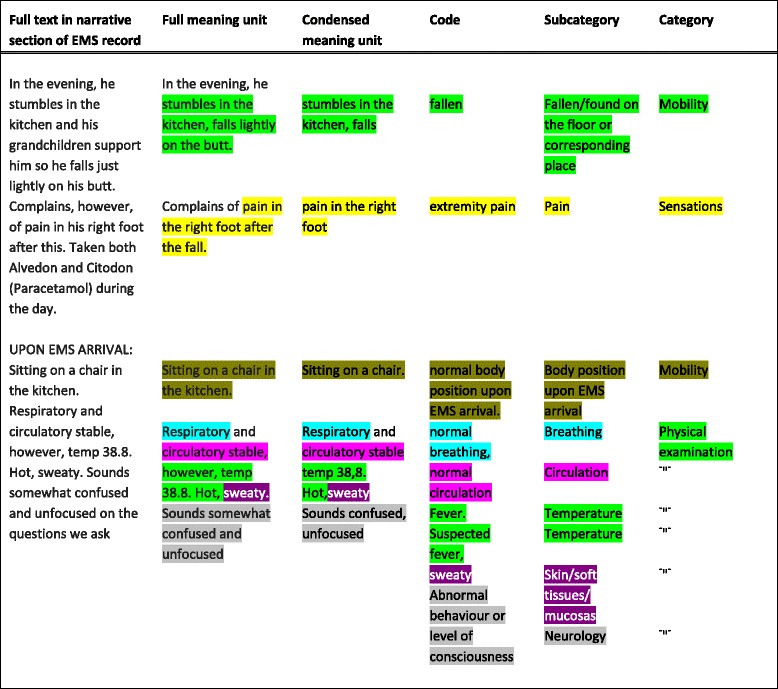
Example of the content analysis of septic patients arriving by EMS and admitted during 2012. EMS = Emergency Medical Services

The full text was read several times and full meaning units were chosen. Text irrelevant to the aim of the study was excluded. Full meaning units were condensed into shorter, condensed meaning units when possible. However, the text of EMS records is frequently brief and it was not always possible to condense it further. As a third step representative codes were identified. These codes were grouped into subcategories which were abstracted into categories [23] (see Fig. 2, Example of the content analysis of patients admitted during 2012). Codes and subcategories (and combinations of such) identified in the content analysis are from now on referred to as “keywords”, to better illustrate the aim of the study.

#### 2. Quantification of keywords among patients admitted during 2013

For the quantification of keywords identified in the content analysis of patients admitted during 2012, the narrative section of EMS records from septic patients admitted during 2013 was analyzed. To describe and quantify clinically relevant keywords, the expressions “primary” and “combined” keywords were introduced. Primary keywords were codes and subcategories derived from the content analysis while combined keywords consist of several primary or combined keywords. Combined keywords were created in order to condense primary keywords so as to make possible for potential use in a future screening tool. Keywords related to septic patients’ symptom presentation were presented separately and defined as keywords that describe the patient’s or bystanders’ experience of the disease i.e., symptom. Frequency tables and cross tables were created using SPSS software (SPSS, Version 22, IBM Company, Chicago, IL, USA, statistical software) and prevalence of the documented keyword is presented as number and percent.

In-hospital mortality within subgroups presenting with various keywords related to symptom presentation was analyzed. Finally, the prevalence of keywords related to septic patients’ symptom presentation was compared between age categories, survivors and deceased, and between patients with severe and non-severe sepsis, using Fischer’s exact test. Differences in categorical variables between patients from 2012 and 2013 were analyzed using Fischer’s exact test and Mann Whitney *U* test was used to analyze differences in numeric variables (age). *P*-values <0.05 were considered statistically significant.

### Ethical approval

Stockholm Regional Ethical Review Board approval was obtained for this study and a waiver of informed consent was granted.

## Results

Eighty patients admitted during 2012 were included in the content analysis. To include 80 EMS records, we needed to analyze 85 EMS records, in turn selected through the maximum variation sampling method [26]. Five patients of the 85 analyzed were excluded due to exclusion criteria as illustrated in Fig. 1. Three hundred fifty-nine patients admitted during 2012 were included in the quantification of keywords. See Fig. 1.

Characteristics of the study patients are presented in Table 1.

**Table 1:** Characteristics of patients in the content analysis admitted during 2012 and patients in the quantification of keywords admitted during 2013. Characteristics of 80 septic patients admitted 2012, included in the content analysis, and 359 septic patients admitted 2013, included in the quantification of keywords^a^

	80 patients in the content analysis admitted during 2012^b^		359 patients in the quantification of keywords admitted during 2013^b^	
Variable	Median (IQR)	Number (%)	Median (IQR)	Number (%)
Age, yr	73 (63–84)	80/80 (100.0)	78 (68–86)	359/359 (100.0)
Gender				
-male		44/80 (55.0)		198/359 (55.2)
Severe sepsis		48/77^c^ (62.3)		203/352^c^ (57.7)
Substance abuse^d^		5/80 (6.3)		22/359 (6.1)
In-hospital mortality				
-total population		18/80 (22.5)		94/359 (26.2)
-severe sepsis		11/48 (22.9)		73/203 (36.0)
-non-severe sepsis		4/29 (13.8)		19/149 (12.8)
EMS clinical judgment sepsis		11 (13.8)		68 (18.9)

IQR = Interquartil range, EMS = Emergency Medical Services

^a^Primary keywords (codes and subcategories derived from the content analysis of septic patients arriving by EMS and admitted to Södersjukhuset during 2012) or combined keywords (consisting of several primary or combined keywords)

^b^There was a significant difference in age between the two groups (*P*-value 0.03). No other significant differences in characteristics were observed. 2-sided *P*-values were calculated by Fischer’s exact test for categorical variables and by Mann Whitney *U* test for numerical variables

^c^Number of patients with enough documented information to determine whether severe sepsis or not

^d^Defined as drug abuse, alcohol overconsumption and all other terms indicating substance abuse such as “lives in a home for addicts”, “patient at an outdoor clinic for substance abuse”

### 1. Content analysis of patients admitted during 2012

Five categories including 22 subcategories were identified in the content analysis of patients admitted during 2012 (Additional file 2).

### 2. Quantification of keywords among patients admitted during 2013

The overall prevalence of the primary and combined keywords is presented in Additional files 3 and 4, respectively.

### Primary outcomes

The prevalence of keywords related to septic patients’ symptom presentation according to EMS documentation, among 359 septic patients admitted during 2013, is presented in Table 2. Seven keywords related to symptom presentation had a prevalence exceeding 20% of the septic patients: abnormal/ suspected abnormal temperature (64.1.%), pain (38.4%), acute altered mental status (38.2%), weakness of the legs (35.1%), breathing difficulties (30.4%), loss of energy (26.2%) and gastrointestinal symptoms (24.0%) (Table 2).

**Table 2:** Prevalence of keywords^a^ related to septic patients’ symptom presentation. Prevalence of keywords^a^ related to septic patients’ symptom presentation, according to EMS documentation, among 359 septic patients arriving by EMS and admitted to Södersjukhuset through the ED during 2013 and in-hospital mortality in relation to these keywords

Order	Keyword^**a**^	Prevalence		In-hospital mortality	
		Number of total 359 patients	Percent (%) and 95% CI	Number of all patients with the keyword present/ documented	Percent (%) and 95% CI Top-5 highest mortality are numbered^1–5^
1	**Abnormal, or suspected abnormal temperature**	230	64.1 (58.9–69.0)	48/230	20.9 (15.8–26.7)
	**-Confirmed or suspected fever** Fever defined as statement fever or statement temperature >38°[41] OR suspected fever defined as statement feeling hot/warm, increasing temperature or similar expressions	210	58.5 (53.2–63.6)	39/210	18.6 (13.6–24.5)
	-Shivering	58	16.2 (12.5–20.4)	6/58	10.3 (3.9–21.2)
	-Hypothermia Hypothermia defined as statement hypothermia or “very low temp” or statement temperature <36°[41]	10	2.8 (1.3–5.1)	8/10	80.0^1^ (44.4–97.5)
2	**Pain** Abdominal, extremity, back, undefined, urinary tract, joint, chest, general, headache, throat, wound, painful muscle cramp, positive Pasternatsy’s sign (costovertebral angle tenderness)	138	38.4 (33.4–43.7)	28/138	20.3 (13.9–28.0)
3	**Acute altered mental status** Abnormal behaviour or level of consciousness (excluding previously known dementia or mental retardation without statement worse) OR abnormal verbal response defined as no/decreased verbal response [25]	137	38.2 (33.1–43.4)	51/137	37.2^4^ (29.1–45.9)
4	**Weakness of the legs**	126	35.1 (30.2–40.3)	27/126	21.4 (14.6–29.6)
	**-Decreased ability to stand or walk** including need to carry/lift the patient	98	27.3 (22.8–32.2)	19/98	19.4 (12.1–28.6)
	**-Fallen/found on the floor** or corresponding place	57	15.9 (12.3–20.1)	13/57	22.8 (12.7–35.8)
5	Breathing difficulties	109	30.4 (25.6–35.4)	39/109	35.8^5^ (26.8–45.5)
6	Loss of energy Defined as fatigue, weakness, faintness or similar expressions	94	26.2 (21.7–31.1)	24/94	25.5 (17.1–35.6)
7	**Gastrointestinal symptoms**	86	24.0 (19.6–28.7)	21/86	24.4 (15.8–34.9)
	-Vomiting	58	16.2 (12.5–20.4)	11/58	19.0 (9.9–31.4)
	-Diarrhoea	35	9.7 (6.9–13.3)	8/35	22.9 (10.4–40.1)
8	**Abnormal urination** ^b^	58	16.2 (12.5–20.4)	13/58	22.4 (12.5–35.3)
	-Decreased urinary volumes	12	3.3 (1.7–5.8)	7/12	58.3^2^ (27.7–84.8)
9	Reduced intake of food, fluid or oral medicines	47	13.1 (9.8–17.0)	18/47	38.3^3^ (24.5–53.6)
10	Nausea	36	10.0 (7.1–13.6)	5/36	13.9 (4.7–29.5)
11	Malaise Defined as expressions such as feeling sick, feeling bad, not feeling well and similar expressions	19	5.3 (3.2–8.1)	3/19	15.8 (3.4–39.6)
12	**Mood change** Anxiety or fear OR feeling of depression	18	5.0 (3.0–7.8)	4/18	22.2 (6.4–47.6)
13	Dizziness	14	3.9 (2.1–6.5)	1/14	7.1 (0.2–33.9)
14	Fainted but now awake	10	2.8 (1.3–5.1)	2/10	20.0 (2.5–55.6)

EMS = Emergency Medical Services. CI = Confidence Interval

^a^Primary keywords (codes and subcategories derived from the content analysis of septic patients arriving by EMS and admitted to Södersjukhuset during 2012) or combined keywords (consisting of several primary or combined keywords). Combined keywords are bolded, primary keywords are not. For combined keywords, the included primary or combined keywords are presented in descending order beneath the name of the keyword

^b^Abnormal urination defined as hematuria without trauma, bad smelling or cloudy urine, increased frequency of urination OR urinary tract pain OR decreased urinary volumes OR dysfunction of urinary catheters defined as obstruction/leakage/problematic urinary catheters including nefrostomias

### Secondary outcomes

The in-hospital mortality in relation to keywords reflecting symptom presentation is presented in Table 2.

The highest in-hospital mortality was observed among patients with documented hypothermia (80.0%), decreased urinary volumes (58.3%), reduced intake of food, fluid or oral medicines (38.3%), history of acute altered mental status (37.2%) and breathing difficulties (35.8%) (Table 2).

Distribution of keywords among subcategories of septic patients is presented in Additional files 5, 6 and 7.

Weakness of the legs was significantly more frequent in the oldest age category (43.8 vs 26.1%, *p*-value 0.02) as compared with patients below 65 years of age (Additional file 5).

Survivors had a higher prevalence of EMS documented abnormal, or suspected abnormal temperature (68.7 vs 51.1%, *p*-value 0.003) and shivering (19.6 vs 6.4%, *p*-value 0.002) as compared with deceased (Additional file 6). Deceased had a higher prevalence of EMS documented hypothermia (8.5 vs 0.8%, *p*-value <0.001), acute altered mental status (54.3 vs 32.5%, *p*-value <0.001), breathing difficulties (41.5 vs 26.4%, *p*-value 0.009) and decreased urinary volumes (7.4 vs 1.9%, *p*-value 0.02) (Additional file 6), as compared with survivors.

EMS documentation of hypothermia (4.9 vs 0.0%, *p*-value 0.006), acute altered mental status (67.5 vs 0%, *p*-value <0.001) and reduced intake of food, fluid or oral medicines (16.7 vs 8.7%, *p*-value 0.04) was significantly more frequent among patients with severe sepsis compared to among those with non-severe sepsis (Additional file 7).

Documented pain (49.7 vs 29.6%, *p*-value <0.001) and nausea (14.1 vs 6.9%, *p*-value 0.03) were significantly more frequent among patients with non-severe sepsis compared to among those with severe sepsis (Additional file 7).

## Discussion

The current study identified keywords related to septic patients’ presentation according to EMS documentation, using a mixed methods approach. The most frequently documented keywords related to patients’ symptom presentation were: abnormal, or suspected abnormal temperature, pain, acute altered mental status, weakness of the legs, breathing difficulties, loss of energy and gastrointestinal symptoms such as vomiting and diarrhoea.

Certain presentations were associated with increased in-hospital mortality and the distribution of keywords in relation to age categories, survivors/ deceased and severe/ non-severe sepsis varied.

Keywords related to symptom presentation are not included in the existing screening tools for sepsis identification within emergency care [17, 18], which should be reconsidered. However, before this is done, prospective studies evaluating the sensitivity and specificity of these keywords needs to be evaluated.

Almost all patients that presented with the most common combined keyword; abnormal or suspected abnormal temperature had fever, while hypothermia was in general rare but more common among patients with severe sepsis (Table 2 and Additional file 7). Despite fever being frequently documented as a symptom in the EMS records; approximately one third of the patients lacked this finding. This observation is consistent with a previous study of bacteraemic ED patients by Lindvig et al. [34], showing that 34.1% of bacteraemic patients had a normal temperature recorded at ED arrival.

Pain was frequently documented. The most common locations were the abdomen, extremity, back and urinary tract. The location often reflected the site of the original infection but general flu-like muscular pain was also common, in accordance to previous literature, describing diffuse pain as frequent [19].

The combined keyword acute altered mental status, represents primary keywords ranging from altered behaviour to the deepest level of non-responsiveness and may reflect sepsis-associated encephalopathy (SAE) [35, 36], known to affect up to 70% of patients with severe sepsis [37]. It could be described by the patient in terms such as “feeling confused” or “feeling sleepy” or not remembering events in the last days, and by relatives as an observed disorientation, a lack of attention or an inability to verbally response [19].

Weakness of the legs was another common symptom presentation. This has, to the best of our knowledge, not previously been described for septic patients in the prehospital setting. However, previous studies have indicated that sepsis induces a myopathy characterized by reduced muscle force-generating capacity, and loss of muscle mass [38], and weakness of the legs is interpreted as an expression of this pathophysiology.

Breathing difficulties were frequently documented. Interestingly, only 39% of the patients with documented breathing difficulties had a pulmonary origin of the underlying infection, indicating that breathing difficulties are frequent in sepsis with a focus other than the lung. This may in turn suggest that the presentation of breathing difficulties is part of a systemic pathophysiological response to the underlying infection, which may include an anaerobic metabolism and metabolic acidosis.

In-hospital mortality varied in relation to the documented symptom presentation. The highest mortality rates were observed among patients with documentation of hypothermia, reduced urinary volumes and reduced intake of food or fluid. Interestingly, the mortality rate among patients presenting with these presentations exceeded that of patients presenting with keywords traditionally included in the definition of severe sepsis such as acute altered mental status. However, these findings need to be replicated in larger cohorts.

The documented presentations varied between age categories which may reflect a variation in the physiological response to an infection relating to age. However, it may also reflect that health care personnel direct their questions differently when encountering elderly patients, focusing on more basic functions e.g., food/fluid intake and whether they can stand and walk.

Finally, presentations differed between survivors and deceased. Known or suspected fever and shivering were more frequently documented among survivors which may indicate that these patterns reflect an appropriate immunological response or possibly a protective effect per se. This is consistent with previous studies demonstrating a decreased mortality in septic patients with moderate fever [39].

### Limitations

The analysis of sepsis presentation was based on EMS documentation which is associated with inherent restrictions. Documentation can be affected by many factors e.g., what EMS ask the patient, the patient’s ability to explain his/her experience and the presence of relatives who may or may not be able to describe the situation at hand. It is, as described above, not always possible to discern the origin of the documented information. The EMS records present the symptoms as documented by EMS personnel. To perform open interviews with septic patients would be an alternative approach to explore sepsis symptom presentation. However, interviews in the ambulance would be difficult to perform for logistical reasons. In addition there would be a bias towards less sick patients due to the most sick septic patients being unable to participate in an interview. Moreover, EMS personnel have been shown to have difficulties identifying septic patients [25], which would lead to an inclusion bias. Furthermore, a third of the septic patients present with altered mental status, which would impair their ability to participate in interviews in the acute setting and affect their recall if the interview would be performed in retrospect.

Since the EMS records are brief and often lack detail, there is a risk that not all possible keywords are documented. However, even if the true prevalence of various keywords is expected to be higher than that documented, the relative proportions between the keywords are assumed to be similar.

Inclusion based on ICD codes has been used in several previous studies [27, 40] and is the only reasonable way for database searches, but can be questioned as it is well known that diagnostic coding is a problem [41], and consistently underestimates the incidence [42]. Hence, assumedly more patients with sepsis were admitted by EMS and cared for in-hospital during the study period but discharged with ICD codes other than those compatible with sepsis, e.g., those indicating the focus of infection i.e., pneumonia or meningitis instead of sepsis. Inclusion by the means of ICD code could potentially entail a selection of more sick patients, i.e., a higher proportion of severe sepsis as well as patients with symptoms more typical of the common picture of sepsis, e.g., fever and hemodynamic instability since these patients may be more readily identified in the clinical setting. Hence, the inclusion based on ICD codes may limit the generalizability or transferability [22] (the corresponding term within qualitative research) of our results to all possible septic patients.

Furthermore, the creation of keywords may have been influenced by the preconceptions of the authors, which is inherent in all qualitative analyzes, and the creation of exclusive subcategories was sometimes difficult as many of the complaints resembled each other. The authors have different backgrounds and met regularly to ensure trustworthiness and a consistent approach to analysis of the data.

The mixed methods approach [20, 21], starting with an inductive content analysis [43, 44], is used to explore previously unstudied areas. Hence, the current study should be viewed as the necessary first step in upcoming studies aiming to identify parameters with a high predictive value with respect to sepsis identification. As a first step it was necessary to identify keywords which could be analyzed in prospective studies and compared between septic and non-septic patients. The keywords in the current study are most likely not pathognomonic for sepsis. Moreover, it is unlikely that there are unique keywords pathognomonic for sepsis as the presentation is so diverse, but we do believe in the predictive value of combinations of keywords related to presentation and possibly together with other parameters measurable in the ambulance.

The frequency of keywords was sometimes associated with broad 95% Confidence Intervals (CIs). Especially in the subgroup analyses presented in Additional files 5, 6 and 7 the CIs indicate that larger study samples would be required for an increased precision.

Finally, the identified keywords were those documented by EMS within a cohort of septic patients admitted to the ED of Södersjukhuset by EMS and discharged with ICD code sepsis. It is possible that septic patients discharged with more organ specific ICD codes / arriving by other means than EMS, as well as septic patients in other settings could present with other symptoms. Hence the results of the current study may not be generalizable / transferable to other settings. Prospective studies are needed to analyze whether the identified keywords are representative for septic patients in general and to understand their predictive value. We suggest that it is necessary to include keywords in sepsis screening tools, however, which specific keywords or combinations thereof remain to be studied.

## Conclusions

Keywords related to patients’ symptom presentation recurred in EMS records of septic patients in the prehospital setting, so that a pattern was discernible. In addition, certain symptom presentations were associated with increased in-hospital mortality. This information is not included in current sepsis screening tools and keywords related to patients’ symptom presentation could potentially be used to increase the identification of sepsis, and possibly identify high-risk patients. However, as a first step, the specificity of these keywords, with respect to sepsis, needs to be examined.

## Additional files

Additional file 1: Definition of severe sepsis. (DOC 28 kb)

Additional file 2: Five categories and 22 included subcategories identified in the content analysis of patients admitted 2012. (DOC 31 kb)

Additional file 3: Prevalence of primary keywords* among septic patients arriving by EMS and admitted during 2013. (DOC 107 kb)

Additional file 4: Prevalence of combined keywords* among septic patients arriving by EMS and admitted during 2013. (DOC 49 kb)

Additional file 5: Prevalence of combined keywords* among septic patients arriving by EMS and admitted during 2013. (DOC 49 kb)

Additional file 6: A comparison of the prevalence of keywords* related to symptom presentation between survivors and deceased†. (DOC 69 kb)

Additional file 7: A comparison of the prevalence of keywords* related to symptom presentation between severe/ non-severe sepsis. (DOC 69 kb)

## Abbreviations

ED: Emergency Department
EMS: Emergency Medical Services
HCAI: Healthcare-Associated Infection
ICD-10-code: International Classification of Diseases, Tenth Revision
SAE: Sepsis-Associated Encephalopathy
SIRS: Systemic Inflammatory Response Syndrome
SPSS: Statistical Package for the Social Sciences
