# Supplementary material for: Presentations of adult septic patients in the prehospital setting as recorded by emergency medical services: a mixed methods analysis
Source: Scand J Trauma Resusc Emerg Med. 2017 Mar 3;25:23. doi: 10.1186/s13049-017-0367-z (PMC5439232; doi:10.1186/s13049-017-0367-z)
Supplement: Supplementary file 1 — Definition of severe sepsis. (DOC 28 kb) [file 13049_2017_367_MOESM1_ESM.doc]

**Additional file 1. Definition of severe sepsis**

Severe sepsis was defined as fulfillment of one or more of the following criteria during EMS transport; systolic blood pressure <90 mmHg [1,2] or an EMS statement of a non-measurable blood pressure [2], oxygen saturation of ≤86% if the lung was not the focus of infection or oxygen saturation ≤78% if the lung was focus of infection [2,3], acute altered mental status [2,3], mottling [1,2] or cardiopulmonary arrest due to sepsis during EMS transport [2] (but admitted alive to in-hospital care).

*References:*

*1. Levy MM, Fink MP, Marshall JC, et al. 2001 SCCM/ESICM/ACCP/ATS/SIS International Sepsis Definitions Conference. Critical care medicine. 2003;31:1250-1256.*

*2. Wallgren UM, Castren M, Svensson AE, et al. Identification of adult septic patients in the prehospital setting: a comparison of two screening tools and clinical judgment. Eur J Emerg Med. Sep 30 2013.*

*3. Svenska Infektionsläkarföreningen. [Swedish guidelines for treatment of severe sepsis and septic shock]. 2012. (http://www.infektion.net/kunder/infektion/kunder/infektion/sites/default/files/6/Vardprogram_svarsepsis__2012.pdf )*
