# Supplementary material for: Presentations of adult septic patients in the prehospital setting as recorded by emergency medical services: a mixed methods analysis
Source: Scand J Trauma Resusc Emerg Med. 2017 Mar 3;25:23. doi: 10.1186/s13049-017-0367-z (PMC5439232; doi:10.1186/s13049-017-0367-z)
Supplement: Supplementary file 2 — Five categories and 22 included subcategories identified in the content analysis of patients admitted 2012. (DOC 31 kb) [file 13049_2017_367_MOESM2_ESM.doc]

**Additional file 2. Five categories and 22 included subcategories identified in the content analysis of patients admitted 2012.**

| **Categories** | **Included subcategories** |
| --- | --- |
| **PHYSICAL EXAMINATION** | Breathing/respiration  Circulation  Temperature  General condition  Neurology  Skin/soft tissues/mucous membranes  Soiled patient (wetted from urine or stool or bloodstained) |
| **SENSATIONS** | Pain  Nausea  Loss of energy  Malaise  Dizziness  Mood change |
| **MOBILITY** | Body position upon EMS arrival  Ability to walk or stand  Fallen/ found on the floor or corresponding place  Additional problems related to mobility (expresses e.g. stiffness when trying to move arms, inability to sit or inability to squeeze the investigator´s hand) |
| **ELIMINATION** | Gastrointestinal function  Urination |
| **ADDITIONAL INFORMATION ASSOCIATED TO SEPSIS PRESENTATION** | Temporal deterioration  Decreased intake of food, fluid or oral medicines  Risk factors for sepsis |
| The terms are further explained in the following tables. | |

**Five categories and 22 included subcategories identified in the content analysis of 80 EMS records for septic patients arriving by EMS and admitted to Södersjukhuset during 2012.**
