# Supplementary material for: Presentations of adult septic patients in the prehospital setting as recorded by emergency medical services: a mixed methods analysis
Source: Scand J Trauma Resusc Emerg Med. 2017 Mar 3;25:23. doi: 10.1186/s13049-017-0367-z (PMC5439232; doi:10.1186/s13049-017-0367-z)
Supplement: Supplementary file 3 — Prevalence of primary keywords* among septic patients arriving by EMS and admitted during 2013. (DOC 107 kb) [file 13049_2017_367_MOESM3_ESM.doc]

**Additional file 3. Prevalence of primary keywords* among septic patients arriving by EMS and admitted during 2013.**

| **Order** | **Primary keyword ⃰** | **Number of total 359 patients** | **Percent (%) and 95% CI** |
| --- | --- | --- | --- |
| **1** | **Supine patient position upon EMS arrival** Separate subheading “upon EMS arrival” in the narrative part of the EMS record | 212 | 66.9 (61.4-72.0) |
| **2** | **Confirmed fever** Statement fever or statement temperature >38˚[1] | 176 | 49.0 (43.7-54.3) |
| **3** | **Tachypnea** Statement tachypnea, rapid breathing, high respiratory rate, respiratory rate >20 [1], or similar expressions | 128 | 35.7 (30.7-40.9) |
| **4** | **Abnormal behaviour or level of consciousness**  excluding abnormal verbal response | 121 | 33.7 (28.8-38.9) |
| **5** | **Tachycardia** Statement heart rate >90/min [1] or expressions such as rapid heart rate, rapid pulse or similar expressions | 111 | 30.9 (26.2-36.0) |
| **6** | **Breathing difficulties** Statement difficulties to breath, dyspnea, shortness of breath, shallow breathing or similar expressions | 109 | 30.4 (25.6-35.4) |
| **7** | **Decreased ability to stand or walk**  Including need to carry/lift the patient | 98 | 27.3 (22.8-32.2) |
| **8** | **Loss of energy** Defined as fatigue, weakness, faintness or similar expressions | 94 | 26.2 (21.7-31.1) |
| **9** | **Low blood pressure** Statement systolic blood pressure ≤90 mmHg [1] | 82 | 22.8 (18.6-27.5) |
| **10** | **Suspected fever** Defined as statement feeling hot/warm, increasing temperature or similar expressions | 80 | 22.3 (18.1-26.9) |
| **11** | **Low oxygen saturation** Defined as statement oxygen saturation <90% (reference clinical praxis for need of oxygen treatment) | 77 | 21.4 (17.3-26.1) |
| **12** | **Temporal deterioration** Stated deterioration or expressions describing a temporal change | 75 | 20.9 (16.8-25.5) |
| **13** | **Abnormal verbal response** Defined as no/decreased/changed verbal response | 71 | 19.8 (15.8-24.3) |
| **14** | **Shivering** | 58 | 16.2 (12.5-20.4) |
| **15** | **Vomiting** | 58 | 16.2 (12.5-20.4) |
| **16** | **Explicitly expressed difficulties to stand or walk** | 55 | 15.3 (11.8-19.5) |
| **17** | **Pale** | 53 | 14.8 (11.3-18.9) |
| **18** | **Decreased general condition** Including expressions such as poor general condition, affected general condition | 48 | 13.4 (10.0-17.3) |
| **19** | **Non-measurable circulatory variables** | 48 | 13.4 (10.0-17.3) |
| **20** | **Reduced intake of food, fluids or oral medicines** Including reduced/no appetite | 47 | 13.1 (9.8-17.0) |
| **21** | **Fallen** | 45 | 12.6 (9.3-16.5) |
| **22** | **Airway secretions** Including expectorations, crackles and similar expressions | 45 | 12.5 (9.3-16.4) |
| **23** | **Oedema/Swelling** | 45 | 12.5 (9.3-16.4) |
| **24** | **Abdominal pain** | 44 | 12.3 (9.0-16.1 |
| **25** | **Known ongoing or recent infection** | 41 | 11.4 (8.3-15.2) |
| **26** | **Abnormal urination, original version** Defined as hematuria without trauma, bad smelling or cloudy urine, increased frequency of urination | 38 | 10.6 (7.6-14.2) |
| **27** | **Wounds or wound infection** | 36 | 10.0 (7.1-13.6) |
| **28** | **Nausea** | 36 | 10.0 (7.1-13.6) |
| **29** | **Diarrhoea** | 35 | 9.7 (6.9-13.3) |
| **30** | **Current antibiotic treatment** | 34 | 9.5 (6.6-13.0) |
| **31** | **Recent invasive procedures** Including IV drug abuse, surgical and urological procedures, new IV or urinary catheters | 31 | 8.6 (5.9-12.0) |
| **32** | **Focal neurological findings** | 27 | 7.5 (5.0-10.8) |
| **33** | **Sweaty** | 26 | 7.2 (4.8-10.4) |
| **34** | **Extremity pain** | 26 | 7.2 (4.8-10.4) |
| **35** | **Weak pulse or difficulties to palpate the pulse** | 25 | 7.0 (4.6-10.1) |
| **36** | **Remained lying or sitting** Statement of being remained sitting or lying in an abnormal way | 23 | 6.4 (4.1-9.5) |
| **37** | **Substance abuse** Defined as drug abuse, alcohol overconsumption and all other terms indicating substance abuse such as “lives in a home for addicts”, “patient at an outdoor clinic for substance abuse” | 22 | 6.1 (3.9-9.1) |
| **38** | **Back pain** | 22 | 6.1 (3.9-9.1) |
| **39** | **Non-measurable breathing variables** | 22 | 6.1 (3.9-9.1) |
| **40** | **Found on the floor** or corresponding place | 22 | 6.1 (3.9-9.1) |
| **41** | **Peripheral coldness** | 20 | 5.6 (3.4-8.5) |
| **42** | **Cyanosis** Including blue fingers/nails/lips/toes | 20 | 5.6 (3.4-8.5) |
| **43** | **Malaise** Defined as expressions such as feeling sick, feeling bad, not feeling well and similar expressions | 19 | 5.3 (3.2-8.1) |
| **44** | **Cough** | 18 | 5.0 (3.0-7.8) |
| **45** | **Soiled patient** Patient wetted from his/her own urine or stool | 18 | 5.0 (3.0-7.8) |
| **46** | **Anxiety or fear** | 18 | 5.0 (3.0-7.8) |
| **47** | **High blood sugar** Plasma Glucose >12 mmol/L, regardless diabetes or not. | 18 | 5.0 (3.0-7.8) |
| **48** | **Undefined pain** | 17 | 4.7 (2.8-7.5) |
| **49** | **Urinary tract pain** | 16 | 4.5 (2.6-7.1) |
| **50** | **Irregular pulse** | 16 | 4.5 (2.6-7.1) |
| **51** | **Joint pain** | 15 | 4.2 (2.4-6.8) |
| **52** | **Dizziness** | 14 | 3.9 (2.1-6.5) |
| **53** | **Chest pain** | 13 | 3.6 (1.9-6.1) |
| **54** | **Compromised immune system** Chemotherapy or other immunosuppressive treatment | 13 | 3.6 (1.9-6.1) |
| **55** | **High CRP** Taken previous to EMS arrival | 13 | 3.6 (1.9-6.1) |
| **56** | **Reduced urinary volumes** | 12 | 3.3 (1.7-5.8) |
| **57** | **General pain** | 12 | 3.3 (1.7-5.8) |
| **58** | **Obstructive breathing** | 11 | 3.1 (1.5-5.4) |
| **59** | **Redness (of skin)** | 11 | 3.1 (1.5-5.4) |
| **60** | **Hypothermia** Defined as statement hypothermia or “very low temp” or statement temperature <36 [1] | 10 | 2.8 (1.3-5.1) |
| **61** | **Fainting-but now awake** | 10 | 2.8 (1.3-5.1) |
| **62** | **Gastrointestinal bleeding** Including melena, hematemesis, hematochezia | 10 | 2.8 (1.3-5.1) |
| **63** | **Dysfunction of urinary catheters** Including obstruction/leakage/problematic urinary catheters including nefrostomias | 9 | 2.5 (1.2-4.7) |
| **64** | **Dysarthria** Slurred speech (but non-affected level of consciousness) | 8 | 2.2 (1.0-4.3) |
| **65** | **Headache** | 6 | 1.7 (0.6-3.6) |
| **66** | **Seizures** | 6 | 1.7 (0.6-3.6) |
| **67** | **Icterus** | 6 | 1.7 (0.6-3.6) |
| **68** | **Dry mucous membranes (of the mouth)** | 6 | 1.7 (0.6-3.6) |
| **69** | **Reduced amount of stool** | 6 | 1.7 (0.6-3.6) |
| **70** | **Mottling** | 5 | 1.4 (0.5-3.2) |
| **71** | **Bruises or peteckiae** | 5 | 1.4 (0.5-3.2) |
| **72** | **High blood pressure** Statement high blood pressure or statement blood pressure ≥160 mmHg systolic or ≥100 mmHg diastolic [2] | 5 | 1.4 (0.5-3.2) |
| **73** | **Decreased miscellaneous mobility**  Including expressions such as stiffness when trying to move arms, disability to sit or disability to squeeze the investigators hand | 5 | 1.4 (0.5-3.2) |
| **74** | **Positive Pasternatsky´s sign** Costovertebral angle tenderness | 3 | 0.8 (0.2-2.4) |
| **75** | **Cardiac arrest** | 3 | 0.8 (0.2-2.4) |
| **76** | **Change of skin turgor** | 3 | 0.8 (0.2-2.4) |
| **77** | **Bloodstained patient** | 3 | 0.8 (0.2-2.4) |
| **78** | **Exuding skin** | 2 | 0.6 (0.1-2.0) |
| **79** | **Prolonged capillary refill time** | 1 | 0.3 (0.0-1.5) |
| **80** | **Throat pain** | 1 | 0.3 (0.0-1.5) |
| **81** | **Wound pain** | 1 | 0.3 (0.0-1.5) |
| **82** | **Painful muscle cramp** | 1 | 0.3 (0.0-1.5) |
| **83** | **History of positive findings in blood culture** Positive blood culture taken previous to EMS arrival-during a visit to the hospital or by other health care provider but the patient is now at home | 1 | 0.3 (0.0-1.5) |
| **84** | **Chronically compromised breathing** Such as painful conditions or neurological diseases compromising breathing | 1 | 0.3 (0.0-1.5) |
| **85** | **Palpitations** | 0 | 0.0 (0.0-1.0) |
| **86** | **Feeling of depression** | 0 | 0.0 (0.0-1.0) |
| EMS= Emergency Medical Services, CI=Confidence Interval, IV= Intravenous, CRP= C-Reactive Protein  ⃰ Codes and subcategories derived from the content analysis of septic patients arriving by EMS and admitted to Södersjukhuset during 2012.  *References:*  *1. Ljungstrom LR, Steinum O, Brink M, et al. [Diagnosis and diagnostic coding of severe sepsis and septic shock. ICD-10 should be completed with additional codes]. Lakartidningen. 2011;108:276-278.*  *2. Swedish Agency for Health Technology Assessment and Assessment of Social Services, 2004. [Moderate hypertension]. (Accessed 29 February 2016, at* *(http://www.sbu.se/sv/publikationer/SBU-utvarderar/mattligt-forhojt-blodtryck/)* | | | |

**Prevalence of primary keywords ⃰ from the narrative section of EMS records, among 359 septic patients arriving by EMS and admitted to Södersjukhuset during 2013.**
