# Supplementary material for: Presentations of adult septic patients in the prehospital setting as recorded by emergency medical services: a mixed methods analysis
Source: Scand J Trauma Resusc Emerg Med. 2017 Mar 3;25:23. doi: 10.1186/s13049-017-0367-z (PMC5439232; doi:10.1186/s13049-017-0367-z)
Supplement: Supplementary file 4 — Prevalence of combined keywords* among septic patients arriving by EMS and admitted during 2013. (DOC 49 kb) [file 13049_2017_367_MOESM4_ESM.doc]

**Additional file 4. Prevalence of combined keywords ⃰ among septic patients arriving by EMS and admitted during 2013.**

| **Order** | **Combined keyword ⃰** | **Number of total 359 patients** | **Percent (%) and 95% CI** |
| --- | --- | --- | --- |
| **1** | **Abnormal, or suspected abnormal temperature**  In turn including primary keywords† shivering OR hypothermia OR the following combined keywords: | 230 | 64.1 (58.9-69.0) |
|  | **-Confirmed or suspected fever** Fever defined as statement fever or statement temperature >38˚[1] OR suspected fever defined as statement feeling hot/warm, increasing temperature or similar expressions | 210 | 58.5 (53.2-63.6) |
|  | **-Confirmed abnormal temperature (confirmed fever or hypothermia)** Statement fever or statement temperature >38˚[1] OR statement hypothermia or temperature <36˚[1] | 186 | 51.8 (46.5-57.1) |
| **2** | **Abnormal circulation** Weak pulse or difficulties to palpate the pulse, peripheral coldness, cardiac arrest, tachycardia, low blood pressure, prolonged capillary refill time or non-measurable circulatory variables | 193 | 53.8 (48.5-59.0) |
| **3** | **Abnormal breathing** Tachypnea, low oxygen saturation, airway secretions, breathing difficulties, cough, or obstructive breathing | 190 | 52.9 (47.6-58.2) |
| **4** | **Decreased mobility, total** In turn including primary keywords† remained sitting or lying in an abnormal way OR decreased miscellaneous mobility OR the following combined keywords: | 138 | 38.4 (33.4-43.7) |
|  | **-Weakness of the legs** Decreased ability to stand or walk including need to carry/lift the patient OR fallen OR found on the floor or corresponding place | 126 | 35.1 (30.2-40.3) |
|  | **-Fallen or being found on the floor** or corresponding place | 57 | 15.9 (12.3-20.1) |
| **5** | **Pain**  Abdominal, extremity, back, undefined, urinary tract, joint, chest , general, headache, throat, wound, painful muscle cramp, positive Pasternatsy´s sign (costovertebral angle tenderness) | 138 | 38.4 (33.4-43.7) |
| **6** | **Acute altered mental status** Abnormal behaviour or level of consciousness (excluding previously known dementia or mental retardation without statement worse) OR abnormal verbal response defined as no/decreased verbal response [2] | 137 | 38.2 (33.1-43.4) |
| **7** | **Abnormal skin** Pale, wounds or wound infection, sweaty, cyanosis, redness, icterus, mottling, bruises or peteckiae, change of skin turgor, exuding skin | 126 | 35.1 (30.2-40.3) |
| **8** | **Risk factors for sepsis** Known ongoing or recent infection, current antibiotic treatment, recent invasive procedures (see Additional file 5), substance abuse (see see Additional file 5), compromised immune system (see see Additional file 5), chronically compromised breathing (see see Additional file 5) | 110 | 30.6 (25.9-35.7) |
| **9** | **Gastrointestinal symptoms** Vomiting, diarrhoea, reduced amount of stool, gastrointestinal bleeding | 86 | 24.0 (19.6-28.7) |
| **10** | **Abnormal urination** Abnormal urination (such as hematuria without trauma, bad smelling or cloudy urine, increased frequency of urination) OR urinary tract pain OR decreased urinary volumes OR dysfunction of urinary catheters defined as obstruction/leakage/problematic urinary catheters including nefrostomias | 58 | 16.2 (12.5-20.4) |
| **11** | **Abnormal neurology** Focal neurological findings, seizures or dysarthria | 33 | 9.2 (6.4-12.7) |
| **12** | **History of deranged laboratory tests taken previous to EMS arrival** High blood sugar OR history of high CRP OR history of positive findings in blood culture taken previous to EMS arrival | 30 | 8.4 (5.7-11.7) |
| **13** | **Soiled patient** Bloodstained or wetted from stool or urine | 21 | 5.8 (3.7-8.8) |
| **14** | **Mood change** Anxiety or fear OR feeling of depression | 18 | 5.0 (3.0-7.8) |
| EMS= Emergency Medical Services, CI=Confidence Interval, IV= Intravenous, CRP= C-Reactive Protein  ⃰ Consisting of several primary or combined keywords.  †Codes and subcategories derived from the content analysis of septic patients arriving by EMS and admitted to Södersjukhuset during 2012.  *References:*  *1. Ljungstrom LR, Steinum O, Brink M, et al. [Diagnosis and diagnostic coding of severe sepsis and septic shock. ICD-10 should be completed with additional codes]. Lakartidningen. 2011;108:276-278.*  *2. Wallgren UM, Castren M, Svensson AE, et al. Identification of adult septic patients in the prehospital setting: a comparison of two screening tools and clinical judgment. Eur J Emerg Med. Sep 30 2013.* | | | |

**Prevalence of combined keywords ⃰ from the narrative section of EMS records, among 359 septic patients arriving by EMS and admitted to Södersjukhuset during 2013.**
