# Supplementary material for: Presentations of adult septic patients in the prehospital setting as recorded by emergency medical services: a mixed methods analysis
Source: Scand J Trauma Resusc Emerg Med. 2017 Mar 3;25:23. doi: 10.1186/s13049-017-0367-z (PMC5439232; doi:10.1186/s13049-017-0367-z)
Supplement: Supplementary file 6 — A comparison of the prevalence of keywords* related to symptom presentation between survivors and deceased†. (DOC 69 kb) [file 13049_2017_367_MOESM6_ESM.doc]

**Additional file 6. A comparison of the prevalence of keywords ⃰ related to symptom presentation** between survivors and deceased†.

| **Order** | **Keyword** | **Survivors**  **N=265** | | **Deceased†**  **N=94** | | **P-value‡** |
| --- | --- | --- | --- | --- | --- | --- |
|  |  | **Number of total** | **Percent (%) and 95% CI** | **Number of total** | **Percent (%) and 95% CI** |  |
| **1** | **Abnormal, or suspected abnormal temperature** | 182 | 68.7 (62.7-74.2) | 48 | 51.1 (40.5-61.5) | **0.003** |
|  | **-Confirmed or suspected fever** Fever defined as statement fever or statement temperature >38˚[1] OR suspected fever defined as statement feeling hot/warm, increasing temperature or similar expressions | 171 | 64.5 (58.4-70.3) | 39 | 41.5 (31.4-52.1) | **<0.001** |
|  | -Shivering | 52 | 19.6 (15.0-24.9) | 6 | 6.4 (2.4-13.4) | **0.002** |
|  | -Hypothermia Hypothermia defined as statement hypothermia or “very low temp” or statement temperature <36 [1] | 2 | 0.8 (0.1-2.7) | 8 | 8.5 (3.7-16.1) | **<0.001** |
| **2** | **Pain** Abdominal, extremity, back, undefined, urinary tract, joint, chest , general, headache, throat, wound, painful muscle cramp, positive Pasternatsy´s sign (costovertebral angle tenderness) | 110 | 41.5 (35.5-47.7) | 28 | 29.8 (20.8-40.1) | 0.05 |
| **3** | **Acute altered mental status** Abnormal behaviour or level of consciousness (excluding previously known dementia or mental retardation without statement worse) OR abnormal verbal response defined as no/decreased verbal response [2] | 86 | 32.5 (26.9-38.5) | 51 | 54.3 (43.7-64.6) | **<0.001** |
| **4** | **Weakness of the legs** | 99 | 37.4 (31.5-43.5) | 27 | 28.7 (19.9-39.0) | 0.17 |
|  | -Decreased ability to stand or walk including need to carry/lift the patient | 79 | 29.8 (24.4-35.7) | 19 | 20.2 (12.6-29.8) | 0.08 |
|  | -Fallen/found on the floor or corresponding place | 44 | 16.6 (12.3-21.6) | 13 | 13.8 (7.6-22.5) | 0.62 |
| **5** | Breathing difficulties Statement difficulties to breath, dyspnea, shortness of breath, shallow breathing or similar expressions | 70 | 26.4 (21.2-32.2) | 39 | 41.5 (31.4-52.1) | **0.009** |
| **6** | Loss of energy Defined as fatigue, weakness, faintness or similar expressions | 70 | 26.4 (21.2-32.2) | 24 | 25.5 (17.1-35.6) | 1.00 |
| **7** | **Gastrointestinal symptoms** | 65 | 24.5 (19.5-30.2) | 21 | 22.3 (14.4-32.1) | 0.78 |
|  | -Vomiting | 47 | 17.7 (13.3-22.9) | 11 | 11.7 (6.0-20.0) | 0.20 |
|  | -Diarrhoea | 27 | 10.2 (6.8-14.5) | 8 | 8.5 (3.7-16.1) | 0.84 |
| **8** | **Abnormal urination∫** | 45 | 17.0 (12.7-22.1) | 13 | 13.8 (7.6-22.5) | 0.52 |
|  | -Decreased urinary volumes | 5 | 1.9 (0.6-4.3) | 7 | 7.4 (3.0-14.7) | **0.02** |
| **9** | Reduced intake of food, fluid or oral medicines | 29 | 10.9 (7.5-15.3) | 18 | 19.1 (11.8-28.6) | 0.05 |
| **10** | Nausea | 31 | 11.7 (8.1-16.2) | 5 | 5.3 (1.7-12.0) | 0.11 |
| **11** | Malaise Defined as expressions such as feeling sick, feeling bad, not feeling well and similar expressions | 16 | 6.0 (3.5-9.6) | 3 | 3.2 (0.7-9.0) | 0.42 |
| **12** | **Mood change** Anxiety or fear OR feeling of depression | 14 | 5.3 (2.9-8.7) | 4 | 4.3 (1.2-10.5) | 1.00 |
| **13** | Dizziness | 13 | 4.9 (2.6-8.2) | 1 | 1.1 (0.0-5.8) | 0.13 |
| **14** | Fainted but now awake | 8 | 3.0 (1.3-5.9) | 2 | 2.1 (0.3-7.5) | 1.00 |
| EMS= Emergency Medical Services, CI= Confidence Interval.  ⃰ Primary keywords (codes and subcategories derived from the content analysis of septic patients arriving by EMS and admitted to Södersjukhuset during 2012) or combined keywords (consisting of several primary or combined keywords). Combined keywords are bolded, primary keywords are not. For combined keywords, the included primary or combined keywords are presented in descending order beneath the name of the combined keyword.  †in-hospital deceased in accordance with the in-hospital record system.  ‡2-sided P-value calculated by Fischer´s exact test. Significant P-values are bolded.  ∫Abnormal urination defined as hematuria without trauma, bad smelling or cloudy urine, increased frequency of urination OR urinary tract pain OR decreased urinary volumes OR dysfunction of urinary catheters defined as obstruction/leakage/problematic urinary catheters including nefrostomias.  *References:*  *1. Ljungstrom LR, Steinum O, Brink M, et al. [Diagnosis and diagnostic coding of severe sepsis and septic shock. ICD-10 should be completed with additional codes]. Lakartidningen. 2011;108:276-278.*  *2. Wallgren UM, Castren M, Svensson AE, et al. Identification of adult septic patients in the prehospital setting: a comparison of two screening tools and clinical judgment. Eur J Emerg Med. Sep 30 2013.* | | | | | | |

**A comparison of the prevalence of keywords ⃰ related to septic patients´ symptom presentation between survivors and deceased†, among 359 septic patients arriving by EMS and admitted to Södersjukhuset during 2013.**
